# Differential Pathogenic Th17 Profile in Mesenteric Lymph Nodes of Crohn's Disease and Ulcerative Colitis Patients

**DOI:** 10.3389/fimmu.2019.01177

**Published:** 2019-05-28

**Authors:** Marwa Bsat, Laurence Chapuy, Manuel Rubio, Ramses Wassef, Carole Richard, Frank Schwenter, Rasmy Loungnarath, Geneviève Soucy, Heena Mehta, Marika Sarfati

**Affiliations:** ^1^Immunoregulation Laboratory, Centre de Recherche du CHUM (CRCHUM), Montreal, QC, Canada; ^2^Department of Gastrointestinal Surgery, CHUM, Montreal, QC, Canada; ^3^Department of Pathology, CHUM, Montreal, QC, Canada

**Keywords:** Th17 cells, mesenteric lymph nodes, inflammatory bowel disease, human, plasticity

## Abstract

The drug targets IL23 and IL12 regulate pathogenicity and plasticity of intestinal Th17 cells in Crohn's disease (CD) and ulcerative colitis (UC), the two most common inflammatory bowel diseases (IBD). However, studies examining Th17 dysregulation in mesenteric lymph nodes (mLNs) of these patients are rare. We showed that in mLNs, CD could be distinguished from UC by increased frequencies of CCR6^+^CXCR3^−^RORγ^+^Tbet^−^CD4^+^ (Th17) memory T cells enriched in CD62L^low^ effector memory T cells (T_EM_), and their differentially expressed molecular profile. Th17 T_EM_ cells (expressing *IL17A, IL17F, RORC*, and *STAT3*) displayed a higher pathogenic/cytotoxic (*IL23R, IL18RAP*, and *GZMB, CD160, PRF1*) gene signature in CD relative to UC, while non-pathogenic/regulatory genes (*IL9, FOXP3, CTLA4*) were more elevated in UC. In both CD and UC, IL12 but not IL23, augmented IFNγ expression in Th17 T_EM_ and switched their molecular profile toward an ex-Th17 (Th1^*^)-biased transcriptomic signature (increased *IFNG*, and decreased *TCF7, IL17A*), suggesting that Th17 plasticity occurs in mLNs before their recruitment to inflamed colon. We propose that differences observed between Th17 cell frequencies and their molecular profile in CD and UC might have implications in understanding disease pathogenesis, and thus, therapeutic management of patients with IBD.

## Introduction

Lymph nodes (LNs) are the key sites to initiate an effector response and generate memory T cells. However, human lymphoid tissue samples available for research use is quite limited. Recent studies examined several organs of deceased healthy donors, which expanded our knowledge on T cell compartmentalization throughout the body under homeostatic conditions (1, 2). Naïve vs. memory CD4^+^ T cell balance shifts with age, with memory cells gaining numbers in adulthood in mucosal and lymphoid tissues (3). Mesenteric lymph nodes (mLNs) home gut migratory DCs that dictate the type of helper T (Th) responses by driving naïve T cell polarization toward Th1, Th2, Th9, Th17, T follicular helper (Tfh) or regulatory T (T_reg_) cells, each specialized in immunogenic or tolerogenic immune responses (4). Migratory DCs further induce the expression of gut homing receptors such as CCR9 or α4β7 on antigen-specific T cells, which enable their return to the intestine (5). MLNs are thus considered a primary site for generation of mucosal Th responses, including Th17 cells that are important contributors to gut homeostasis. Indeed, an essential role was demonstrated for murine mLNs in the generation of pro-inflammatory IL17A^+^CD4^+^ T cells that are found in the small intestine (6). However, Takebayashi et al. found that absence of mLNs did not affect IL17 cytokine production by CD4^+^ T cells isolated from the colonic lamina propria in murine IBD models (7). Furthermore, it is proposed that Th17 cells are generated in the gut and rarely observed in mLNs and Peyer's patches (8). Studies investigating Th cells in mLNs of patients with inflammatory bowel diseases (IBD) remain scarce (9–12).

Crohn's disease (CD) and ulcerative colitis (UC) are the two most frequent chronic, remitting and relapsing IBD forms (13). Both diseases share common features but are distinct entities with CD developing in the entire gastrointestinal tract and UC in colon and rectum. The immune mechanisms that govern UC and CD disease process include the recruitment of pathogenic Th17 cells in the gut. Pathogenicity of mucosal Th17 cells is not defined by IL17 secretion *per se* but rather by their plastic nature, a hallmark of IBD (14). Th17 conversion to ex-Th17 (Th1^*^) is predominantly controlled by two pro-inflammatory cytokines IL12 and IL23 that share a common p40 chain (15). Yet, the potential contribution of mLNs to the generation of pre-committed pathogenic Th17 cells during intestinal inflammation in CD and UC remains to be investigated. In the present study, we examined the distribution of memory Th17 cells in the mLNs of UC and CD patients, their molecular characteristics, and determined their plasticity in response to IL12 and IL23.

## Materials and Methods

### Human Clinical Samples

MLNs were collected from surgical resections. This study included 25 patients with CD and 9 patients with UC (clinical information is shown in Supplementary Table 1). No histological data or bacterial infections suggested a differential diagnosis.

### Cell Purification and Analysis

MLNs were digested mechanically to obtain cellular suspensions (11). Antibodies used for flow cytometry are listed in Supplementary Table 2. Their respective Fluorescence minus one (FMO) or isotype controls are shown in Supplementary Figure 1. FCS Express 6 (DeNovo Software) or *t*-SNE (*t*-Distributed Stochastic Neighbor Embedding) plugin available in FlowJo version 10.5.3 (FlowJo, LLC) (16) were used for data analysis.

### Cell Sorting and Culture

CD62L^low^CD45RO^+^CD45RA^−^CD25^−^CD8^−^CD4^+^ T cell subsets: CCR6^+^CXCR3^−^, CCR6^+^CXCR3^+^, and CCR6^−^CXCR3^+^ were FACS sorted for functional studies according to the gating strategy depicted in Figure 2A. Transcriptomic studies examined sorted CCR6^+^CXCR3^−^CD62L^low^CD45RO^+^CD45RA^−^CD25^−^CD8^−^CD4^+^ T cells treated in the absence or presence of IL12. Cell isolation was performed using FACS Aria II cell sorter and data were analyzed using FACS Diva 6 (BD Biosciences).

The three purified CD4^+^ T cell subsets were stimulated with anti-CD3/CD28 beads (Miltenyi Biotec) and cultured with or without IL12 (20 ng/ml, R&D system) or IL23 (10 ng/ml, R&D system) for 6 days. Cultures were performed in RPMI 1640 medium supplemented with 10% fetal calf serum and 1% penicillin/streptomycin; 20,000–50,000 cells per well. For intracytoplasmic staining, PMA-ionomycin was added for 6 h in cell cultures and Brefeldin A for the last 3 h, cells were then fixed and stained with CD3 monoclonal antibody followed by intracytoplasmic staining for IL17 and IFNγ.

### NanoString

NanoString was performed at the LDI Molecular Pathology Research Core. RNA was isolated using the NucleoSpin RNA extraction protocol followed by nCounter Low RNA Input Amplification Protocol (nanoString).

Differential gene expression was assessed using the NanoString Human Immunology v2 panel according to the manufacturer's specifications. In brief, amplified RNA was used for Sample Preparation. The samples were then processed with the nCounter Preparation Station to purify the hybridized targets and affix them to the cartridge for imaging using the nCounter Digital Analyzer (CCD camera). Barcodes were counted for each target molecule at High Resolution.

### NanoString Statistical Analysis

The mRNA expression matrix for 583 genes was normalized using a list of house-keeping genes including *ABCF1, ALAS1, EEF1G, G6PD, GUSB, HTPRT1, OAZ1, POLR2A, PPIA, RPL19, TBP, TUBB*. However, it excluded *GAPDH* for having a high expression SD in our dataset. Subsequent PCA analysis revealed that the house-keeping normalized data was primarily clustered by diseases (UC and CD) which is of biological significance. In order to validate the inclusion of a patient covariable in the association model, we performed normalization using the R program (17): R limma (18) and EdgeR (19, 20) library that removed the effect of the patient identity on the PCA expression pattern. The resulting PCA analysis graph showed the samples being clustered by conditions (control and IL12) for which we want to analyze the expression.

A differential expression analysis was done with the R limma package with three contrast matrices:

1. ContUC vs. ContCD (Differential expression analysis between Control samples from UC and CD)
2. IL12CD vs. ContCD (Different expression analysis between IL12 stimulated cell vs. control for CD)
3. IL12UC vs. ContUC (Different expression analysis between IL12 stimulated cell vs. control for UC)

The association model included the contrast sample condition plus a covariate for the patient identity to reflect what was detected on the PCA analysis.

Graphics and visualization of the differential expression analysis metrics where done using the gplots, ggplot2, and beanplot libraries.

### Statistical Analysis

Statistical analysis was performed with Prism version 6 (GraphPad Software). Data were checked for normality using Shapiro-Wilk test and then the appropriate test was applied as indicated. For all tests, 1 symbol means *P* < 0.05, 2 symbols mean *P* < 0.01, and 3 symbols mean *P* < 0.001. Bar graphs are shown as mean ± SEM.

### Study Approval

This study was approved by the Institutional Ethics Research Committee of the Center Hospitalier de l'Université de Montréal (CER-CHUM). The patients provided written consent to the study protocol.

## Results

### Predominance of CCR6^+^CXCR3^−^CD4^+^ T Cells in mLNs of CD When Compared to UC Patients

**Figure 1:**
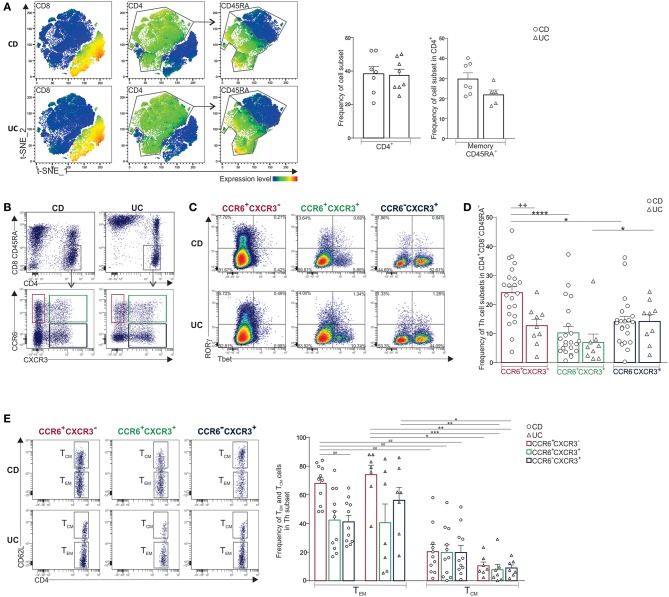
Predominance of CCR6^+^CXCR3-CD4^+^ T cells in mLNs of CD when compared to UC patients. **(A)** CD3^+^ T cells isolated from mLNs of CD and UC patients were concatenated for t-SNE analysis. Feature plots of the indicated antigens (left panels). Frequencies of CD4^+^ and memory CD45RA^−^ T cells (right panels). **(B)** Representative dot plots of CCR6^+^CXCR3^−^, CCR6^+^CXCR3^+^ and CCR6^−^CXCR3^+^ CD4^+^ subsets, **(C)** their expression of RORγ and Tbet, and **(D)** frequencies of indicated Th subsets. **(E)** Representative dot plots and frequencies of T_EM_ (CD62L^low^) and T_CM_ (CD62L^high^) among Th subsets. Unpaired *t*-test (+), Friedmann test followed by Dunn's test (*) and repeated measures one-way ANOVA followed by Tukey's test (#). *P < 0.05, **^++*##*^*P* < 0.01, and ***P < 0.001, ****P < 0.0001.

The human T cell compartment is heterogeneous with variable distribution in different mucosal and lymphoid tissues (3) that is further altered upon inflammation. We investigated whether the distribution of CD4^+^ T cell subsets in inflamed mLNs distinguished CD from UC. The percentage of CD4^+^ T cells and memory CD45RA^−^CD4^+^ T cells was similar in both diseases (Figure 1A). Memory CD4^+^ T cells were next stratified using CCR6 and CXCR3 which are Th17 and Th1-associated markers, respectively (Figure 1B) (21). Accordingly, CCR6^+^CXCR3^−^CD4^+^ T cells expressed RORγ but not Tbet, and conversely, CCR6^−^CXCR3^+^CD4^+^ T cells expressed Tbet but not RORγ (Figure 1C). CCR6^+^CXCR3^+^CD4^+^ T cells co-expressed RORγ and Tbet. Interestingly, the percentage of memory CCR6^+^CXCR3^−^CD4^+^ T cells was significantly higher in CD relative to UC, and additionally, it predominated over both CCR6^−^CXCR3^+^ and CCR6^+^CXCR3^+^ CD4^+^ T cell subsets in CD only (Figure 1D). However, there were no differences between CD and UC in the frequencies of CCR6^−^CXCR3^+^ or CCR6^+^CXCR3^+^ CD4^+^ T cells (Figure 1D). Memory Th cell subsets were further subdivided into CD62L^low^ effector memory (T_EM_) and CD62L^high^ central memory (T_CM_) T cells. As expected, inflamed mLNs comprised more T_EM_ than T_CM_ cells among all Th subsets examined (Figure 1E). However, only in CD the frequencies of CCR6^+^CXCR3^−^ T_EM_ cells were significantly higher than CCR6^−^CXCR3^+^ T_EM_ cells.

Noteworthy, mLN CD4^+^ T cells also comprised minor T subpopulations that were equally distributed in CD and UC, they included T_reg_ (CD25^+^Foxp3^+^), and invariant T cells (Supplementary Figure 2A). The latter comprised MAIT (93–97%) (TCRVα7.2^+^TCRVα4.24^−^), γδ T (1.5–3%) (TCRγδ^+^TCRVα7.2^−^) and iNKT (1.5–3%) (TCRVα7.2^−^TCRVα4.24^+^) cells. T_reg_ and invariant T cell subpopulations were more represented in the CD4^+^ compared to CD8^+^ compartment (Supplementary Figures 2A–C). Within these invariant subpopulations, only 20% of cells expressed CCR6 (Supplementary Figure 2D). Furthermore, T follicular helper cells (Tfh) were detected as rare ICOS^+^CXCR5^+^Ki-67^+^cells in both diseases (Supplementary Figure 2E).

Taken together, CD mLNs comprised more CCR6^+^CXCR3^−^CD4^+^ T cells relative to UC and T_EM_ cells predominated over T_CM_ population.

### MLN Th17 TEM Cells Differentially Expressed a Pathogenic/Cytotoxic Molecular Profile in CD Relative to UC

**Figure 2:**
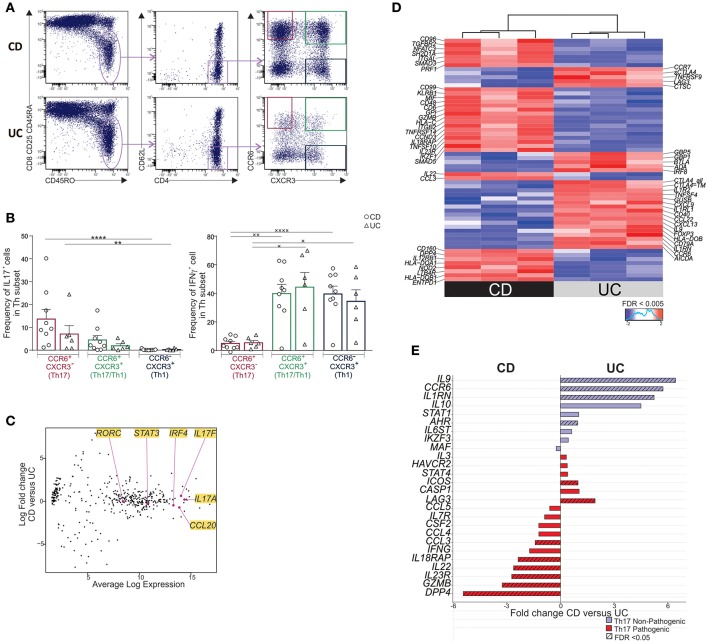
MLN Th17 T_EM_ cells of CD differentially express a pathogenic/cytotoxic molecular profile relative to UC. **(A)** Gating strategy for sorting CCR6 ^±^ CXCR3 ^±^ T_EM_ subsets in mLN. Cells were activated with anti-CD3/anti-CD28-beads for 6 days. On the last day, PMA-ionomycin was added for 6 h and Brefeldin A for the last 3 h. **(B)** Frequencies of IL17 and IFNγ-expressing cells by intracytoplasmic staining in sorted Th T_EM_ subsets. **(C–E)** Cells were activated with anti-CD3/anti-CD28-beads for 6 days. Molecular profiling of mLN Th17 T_EM_ cells in CD (*n* = 3) and UC (*n* = 3) by Nanostring. **(C)** Expression of key Th17 genes in CD vs. UC. **(D)** Heatmap of differentially expressed genes in CD relative to UC (FDR < 0.005). **(E)** Fold change of Th17-associated pathogenic and non-pathogenic genes. Friedmann test followed by Dunn's test (*) and one-way ANOVA followed by Tukey's test (×). ^×^*P* < 0.05, **^,××^*P* < 0.01, and ****^,××××^*P* < 0.0001.

Next, we thought to compare the cytokine and molecular profile of CCR6^+^CXCR3^−^ CD62L^low^CD4^+^ T cells (Th17 T_EM_) in mLNs of UC and CD patients. To this end, mLN Th17 T_EM_, purified as depicted in Figure 2A, expressed high IL17 and low IFNγ while CCR6^+^CXCR3^+^ CD62L^low^CD4^+^ T cells (Th17/Th1 T_EM_) produced both, and CCR6^−^CXCR3^+^CD62L^low^CD4^+^ T (Th1 T_EM_) cells secreted IFNγ only (Figure 2B). However, unlike with unfractionated CD4^+^ T cells (10), no significant differences were noted in the frequencies of IL17 or IFNγ -producing cells in purified Th T_EM_ subsets between CD and UC patients. Th17 T_EM_ identity was further confirmed at the molecular level by equally elevated expression of *IL17A, IL17F, RORC, STAT3*, and *CCL20* in CD and UC (Figure 2C) (4, 14, 22).

Unexpectedly, mLN Th17 T_EM_ subset in CD was distinguished by a set of differentially expressed genes when compared to UC (Figure 2D (FDR < 0.005) and Supplementary Table 3). In fact, *IL23R, CCL3, IL22, DPP4, GZMB*, and *IL18RAP*, reported to be associated with a pathogenic Th17 signature in humans and mice (22–24), were over-expressed in Th17 T_EM_ from CD relative to UC (Figure 2E). *GZMB* and *IL18RAP* along with *PRF1, CSF1, CD160, CXCR6, CD3E, KLRB1* further delineated a pro-inflammatory/cytotoxic Th profile in CD relative to UC (Figures 2D,E and Supplementary Table 3) (25, 26). In contrast, Th17 T_EM_ in UC, when compared to CD, had a greater expression of *IL9, IL10, IL1RN, CTLA4*, and *FOXP3*, genes that are considered non-pathogenic or regulatory (23, 24, 27). Interestingly, augmented *IL9* along with low *CD96* and *DPP4* expression (Figure 2D) observed in UC relative to CD mimics a Th9 pro-inflammatory profile associated with chronic intestinal inflammation in mice (28, 29). Moreover, a Th9-biased profile has also been reported in UC mucosa (30).

In conclusion, Th17 T_EM_ cells are associated with a pathogenic/cytotoxic molecular profile in CD and a non-pathogenic/regulatory profile in UC.

### IL12 Shifts mLN Th17 TEM Cells Toward ex-Th17 (Th1^*^) in CD and UC

**Figure 3:**
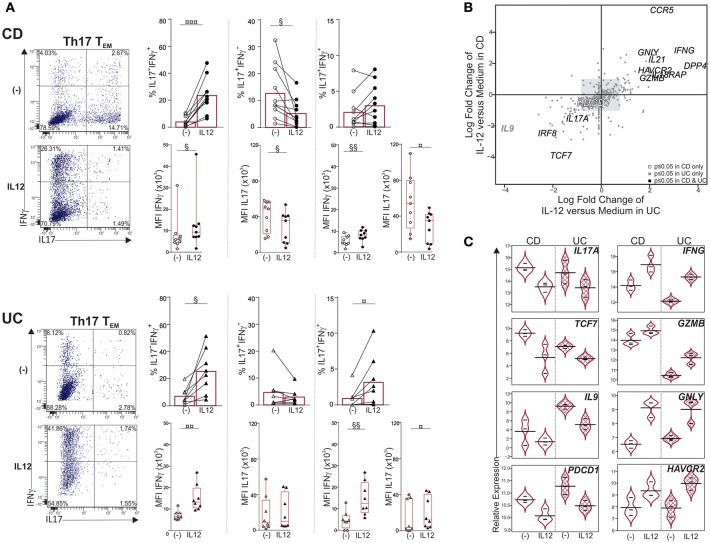
IL12 shifts Th17 T_EM_ cells toward Th1* in mLNs of CD and UC. Sorted mLN Th17 T_EM_ cells were cultured with or without IL12 for 6 days. PMA-ionomycin was added for 6 h and Brefeldin A for the last 3 h. **(A)** Representative dot plots, frequencies and mean fluorescence intensity (MFI) of IL17 and IFNγ. **(B,C)** Molecular profiling of mLN Th17 T_EM_ cells treated with medium or IL12 from CD (*n* = 3) and UC (*n* = 3) by Nanostring. **(B)** Quadrant analysis for differentially expressed genes in Th17 T_EM_, with IL12 treatment relative to medium, in CD vs. UC. **(C)** Violin plots illustrating relative expression of indicated genes. Paired *t*-test (§) or Wilcoxon signed rank test (¤). ^§,¤^*P* < 0.05, ^§§,¤¤^*P* < 0.01, and ^¤¤¤^*P* < 0.001.

IL23 favors Th17 effector function while IL12 down-regulates IL17 and promotes IFNγ expression in circulating and intestinal Th17 cells (21, 23, 31, 32). Furthermore, mucosal pathogenic Th17 cells that contribute to IBD pathogenesis are best defined by their ability to acquire IFNγ, and thus, ultimately switch to Th1^*^ (23). We therefore asked whether Th17 T_EM_ in mLNs could be shifted toward Th1^*^. Th17 T_EM_ exposure to IL12 increased the percentage of IL17^−^IFNγ^+^ cells as well as IFNγ production per cell, as measured by the mean fluorescence intensity (MFI), in both CD and UC (Figure 3A). Frequencies of IL17^+^IFNγ^−^ cells were significantly reduced by IL12 in CD only, further demonstrating a shift of Th17 T_EM_ cells to Th1^*^. In addition, we noticed that IL12 augmented frequencies of IL17^+^IFNγ^+^ cells in 7 out of 9 CD, and 6 out of 8 UC samples. In contrast, IFNγ and IL17 expression was not significantly modified by IL12 in Th17/Th1 T_EM_, and, IFNγ expression was marginally increased in Th1 T_EM_ in UC only (Supplementary Figure 3A).

Furthermore, Th17 conversion to Th1^*^ under the influence of IL12 was associated with *IL17A, TCF7* and *IL9* downregulation while pro-inflammatory and cytotoxic gene expression (*IFNG, IL21, GNLY, DPP4, GZMB)* increased in both CD and UC (Figures 3B,C). *TCF7* downregulation was consistent with the emergence of IL17^−^IFNγ^+^ (Th1^*^) cells and increase of Th1 genes (33, 34). *IFNG* and *HAVCR2* gene expression, which were augmented, best defined Th1-like T cells in colorectal cancer (35), while *PDCD1*, an immune checkpoint inhibitor, was decreased. The gene encoding *IL17F*, that promotes colitis in mice (36), was not inhibited upon IL12 stimulation; reinforcing the concept that IL12 induces the generation of pathogenic Th1^*^ cells in inflamed mLNs. IL12 is likely contributed by mature DCs that accumulate in mLNs of CD (37); these cells also produce IL23 along with IL12 (10). Unlike exposure to IL12, modulation of IL17 and IFNγ expression was unchanged in Th17, Th17/Th1 and Th1 T_EM_ cells in response to IL23 (Supplementary Figure 3B). Failure of IL23 to augment IL17 or IFNγ in Th17 T_EM_ cells was not attributed to absence of IL23 receptor since *IL23R* was expressed in CD, and at higher levels relative to UC (Figure 2D).

Taken together, IL12 but not IL23 promotes plasticity of mLN Th17 T_EM_ cells.

## Discussion

It is established that mucosal Th cells regulate gut homeostasis and inflammation, but few reports examined mLNs of IBD patients. Overall, the present study revealed that CD and UC could be distinguished by examining the frequencies and molecular profile of Th17 cells in mLNs. MLNs in CD were characterized by a predominant Th17 T_EM_ population displaying a pathogenic/cytotoxic gene signature relative to Th17 T_EM_ cells in UC that expressed a profile biased toward regulatory genes. Under IL12 exposure, mLN Th17 T_EM_ cells from both CD and UC shifted toward a Th1^*^ cytokine and molecular profile, suggesting that Th17 plasticity is taking place in this inductive site before T cell homing to gut tissues.

A previous study indicated that frequencies of IL-17^+^ cells are augmented in CD when compared to UC using plastic-coated CD3/CD28 activated unfractionated mLN CD4^+^ T cells (10). We showed here that the proportion of IL17^+^ cells was similar in both diseases using anti-CD3/CD28 beads activated CCR6^+^CXCR3^−^ effector memory CD4^+^ T cells while the expression of IL17 per cell (MFI IL17) tended to augment in CD.

Pathogenicity of Th17 cells is best defined by their capacity to elicit disease after *in vivo* adoptive transfer, their selected gene expression profile and their plastic nature (23, 24, 38). In mice, Th17 cells gradually progress to a pre-Th1 effector phenotype in the LN and, to a Th17/Th1-like effector phenotype in non-lymphoid tissues (39), suggesting that Th17 conversion is already initiated in LN. Indeed in CD mLN, Th17 T_EM_ cells displaying a pathogenic, “pre-Th1” inflammatory gene (*IFNG, HAVCR2, CD26)* profile (22) corroborate the progression observed in mice LN. Furthermore, Th17 T_EM_ cells isolated from the mLN of IBD patients converted to Th1^*^ under the influence of IL12. Th17 program inhibition by IL12, as shown here by decreased *TCF7* expression (34), also depends on *Eomes* up-regulation that inhibits *RORC2* and *IL17A* expression while maintaining *IFNG* (40). Interestingly, *Eomes*-expressing Th1^*^ and Th1 cells have a more stable phenotype and do not up-regulate IL17 under the influence of IL1β, IL6, IL23, and TGFβ Th17-polarizing cytokines (40, 41), consistent with a lack of modulation of IL17 and IFNγ expression observed in CD and UC Th1 T_EM_ cells. The limitation of our study is that EOMES was not part of the nanostring expression matrix.

IL23 expression is required on T cells to trigger colitis, which is associated with IFNγ and IL17 co-expression (38). Furthermore, administration of anti-IL23p19 monoclonal antibody attenuates development of colitis in Abcb1a^−/−^ mice (38, 42). However, Th17 T_EM_ cells from IBD mLNs did not modulate IL17 and IFNγ expression in response to IL23, differing from increased Th17 responses to IL23 observed in human colonic CD4^+^ T cells from CD patients (43). Failure of IL23 to augment IL17 or IFNγ in mLN Th17 T_EM_ cells was not attributed to absence of IL23 receptor since *IL23R* was expressed in CD, and at higher levels relative to UC. These data suggest that tissue-dependent IL23 responsiveness requires additional signals provided by the cytokine milieu and/or environment, which might be absent or low in mLNs. In fact, serum amyloid A proteins 1 and 2 (SAA1/2), secreted by epithelial cells, have been shown to promote robust IL17A production in RORγ^+^ T cells in the mucosa (44). Moreover, a recent study demonstrates that induction of severe chronic remitting/relapsing UC-like colitis in immunocompetent mice requires not only IL23 and pathogenic CD4^+^ T cells in mLNs and colon, but also intestinal dysbiosis (45).

Owing to the importance of IL23/Th17 axis in IBD pathogenesis, it is not surprising that these cells are deemed to be therapeutic targets. However, their inherent plasticity adds difficulty in targeting them directly in inflammatory settings. Antibodies that block IL12 might be suitable targets, as IL12 promotes Th17 plasticity toward pathogenic Th1^*^ in mucosa (21), and as shown here in mLNs of CD and UC. In fact, anti-IL12p40 drugs are part of the therapeutic arsenal of CD, and clinical trials are ongoing in UC patients (46). However, therapeutic efficacy of both anti-IL12p40 and anti-IL23p19 in ameliorating CD argues for a predominant role for IL23 in disease pathogenesis. Hence, anti-IL23p19 is also in clinical trials for UC (46). Collectively, studying the role of immune cells in IBD mLNs warrants further investigation to better understand differences between CD and UC pathogenesis, and thus, open avenues for personalized medicine.

## Data Availability

The nanostring data have been deposited in the Gene expression (GEO) database under accession number GSE131359.

## Ethics Statement

This study was carried out in accordance with the recommendations of CRCHUM with written informed consent from all subjects. All subjects gave written informed consent in accordance with the Declaration of Helsinki. The protocol was approved by the Institutional Ethics Research Committee of the Centre Hospitalier de l'Université de Montréal (CER-CHUM).

## Contribution to the field

While being extensively studied in the mucosa, few studies examined helper T cell subsets in the mesenteric lymph nodes (mLNs) of Crohn's disease (CD) and ulcerative colitis (UC) patients. Lymph nodes are the key sites to initiate an effector response and generate memory T cells, emphasizing the need to investigate immune cells in these lymphoid tissues.

Briefly, we showed that mLNs of CD and UC can be distinguished by frequencies of CXCR3^−^CCR6^+^ Th17 memory T cells, enriched in CD62L^low^ effector memory T cells (T_EM_), and differentially expressed Th17 T_EM_ molecular profile_._ Drug targets IL23 and IL12 regulate the pathogenicity and plasticity of intestinal Th17 cells in IBD. Our data further revealed that IL12, but not IL23, shifted mLN Th17 T_EM_ toward a pathogenic Th1^*^ cytokine and molecular profile in both CD and UC, suggesting that Th17 plasticity is taking place in this inductive site before T cell homing to gut tissues.

Therefore, investigation of CD4^+^ helper T cell subsets in the IBD mLNs, which are not easily accessible for research use, has clear implications in further understanding disease pathogenesis and thus, open avenues for personalized medicine.

## Author Contributions

MB, LC, and MS: Designing research studies. MB, LC, and MR: Conducting experiments. MB, LC, MR, HM, and MS: Acquiring and analyzing data. RW, CR, FS, RL, and GS: Resources. MB, MS, LC, and HM: Writing the manuscript.

### Conflict of Interest Statement

The authors declare that the research was conducted in the absence of any commercial or financial relationships that could be construed as a potential conflict of interest.

## References

[1] Thome JJ Grinshpun B Kumar BV Kubota M Ohmura Y Lerner H. Longterm maintenance of human naive T cells through *in situ* homeostasis in lymphoid tissue sites. Sci Immunol. (2016) 1:eaah6506. 10.1126/sciimmunol.aah6506 28361127 PMC5367636

[2] Kumar BV Connors TJ Farber DL. Human T cell development, localization, and function throughout life. Immunity. (2018) 48:202–13. 10.1016/j.immuni.2018.01.007 29466753 PMC5826622

[3] Senda T Dogra P Granot T Furuhashi K Snyder ME Carpenter DJ. Microanatomical dissection of human intestinal T-cell immunity reveals site-specific changes in gut-associated lymphoid tissues over life. Mucosal Immunol. (2018) 12:378–89. 10.1038/s41385-018-0110-8 30523311 PMC6375790

[4] Stadhouders R Lubberts E Hendriks RW. A cellular and molecular view of T helper 17 cell plasticity in autoimmunity. J Autoimmun. (2018) 87:1–15. 10.1016/j.jaut.2017.12.007 29275836

[5] Iwata M Hirakiyama A Eshima Y Kagechika H Kato C Song SY. Retinoic acid imprints gut-homing specificity on T cells. Immunity. (2004) 21:527–38. 10.1016/j.immuni.2004.08.011 15485630

[6] Kawabe T Suzuki N Yamaki S Sun SL Asao A Okuyama Y. Mesenteric lymph nodes contribute to proinflammatory Th17-cell generation during inflammation of the small intestine in mice. Eur J Immunol. (2016) 46:1119–31. 10.1002/eji.201545907 26887964

[7] Takebayashi K Koboziev I Ostanin DV Gray L Karlsson F Robinson-Jackson SA. Role of the gut-associated and secondary lymphoid tissue in the induction of chronic colitis. Inflamm Bowel Dis. (2011) 17:268–78. 10.1002/ibd.21447 20812332 PMC3072787

[8] Atarashi K Nishimura J Shima T Umesaki Y Yamamoto M Onoue M. ATP drives lamina propria T(H)17 cell differentiation. Nature. (2008) 455:808–12. 10.1038/nature07240 18716618

[9] Saruta M, Yu QT, Avanesyan A, Fleshner PR, Targan SR, Papadakis KA. Phenotype and effector function of CC chemokine receptor 9-expressing lymphocytes in small intestinal Crohn's disease. J Immunol. (2007) 178:3293–300. 10.4049/jimmunol.178.5.3293 17312180

[10] Sakuraba A Sato T Kamada N Kitazume M Sugita A Hibi T. Th1/Th17 immune response is induced by mesenteric lymph node dendritic cells in Crohn's disease. Gastroenterology. (2009) 137:1736–45. 10.1053/j.gastro.2009.07.049 19632232

[11] Baba N Van VQ Wakahara K Rubio M Fortin G Panzini B. CD47 fusion protein targets CD172a+ cells in Crohn's disease and dampens the production of IL-1beta and TNF. J Exp Med. (2013) 210:1251–63. 10.1084/jem.20122037 23669395 PMC3674701

[12] Chapuy L Bsat M Mehta H Rubio M Wakahara K Van VQ. Basophils increase in Crohn disease and ulcerative colitis and favor mesenteric lymph node memory TH17/TH1 response. J Allergy Clin Immunol. (2014) 134:978–981.e971. 10.1016/j.jaci.2014.05.025 24996262

[13] Zhang YZ Li YY. Inflammatory bowel disease: pathogenesis. World J Gastroenterol. (2014) 20:91–9. 10.3748/wjg.v20.i1.91 24415861 PMC3886036

[14] Stockinger B Omenetti S. The dichotomous nature of T helper 17 cells. Nat Rev Immunol. (2017) 17:535–44. 10.1038/nri.2017.50 28555673

[15] Oppmann B Lesley R Blom B Timans JC Xu Y Hunte B. Novel p19 protein engages IL-12p40 to form a cytokine, IL-23, with biological activities similar as well as distinct from IL-12. Immunity. (2000) 13:715–25. 10.1016/S1074-7613(00)00070-4 11114383

[16] Quinn J Duncan S Golden M Swindle M Weiss S Stadnisky M FlowJo Exchange: a means of meeting the computational needs of the flow community. In: CYTO 2015: XXX Congress of the International Society for the Advancement of Cytometry. Glasgow.

[17] R Core Team. R: A Language and Environment for Statistical Computing. Vienna: R Foundation for Statistical Computing (2018). Available online at: https://www.R-project.org/

[18] Ritchie ME Phipson B Wu D Hu Y Law CW Shi W. limma powers differential expression analyses for RNA-sequencing and microarray studies. Nucleic Acids Res. (2015) 43:e47. 10.1093/nar/gkv007 25605792 PMC4402510

[19] Robinson MD McCarthy DJ Smyth GK. edgeR: a Bioconductor package for differential expression analysis of digital gene expression data. Bioinformatics. (2010) 26:139–40. 10.1093/bioinformatics/btp616 19910308 PMC2796818

[20] McCarthy DJ Chen Y Smyth GK. Differential expression analysis of multifactor RNA-Seq experiments with respect to biological variation. Nucleic Acids Res. (2012) 40:4288–97. 10.1093/nar/gks042 22287627 PMC3378882

[21] Annunziato F Cosmi L Santarlasci V Maggi L Liotta F Mazzinghi B. Phenotypic and functional features of human Th17 cells. J Exp Med. (2007) 204:1849–61. 10.1084/jem.20070663 17635957 PMC2118657

[22] Bengsch B Seigel B Flecken T Wolanski J Blum HE Thimme R. Human Th17 cells express high levels of enzymatically active dipeptidylpeptidase IV (CD26). J Immunol. (2012) 188:5438–47. 10.4049/jimmunol.1103801 22539793

[23] Ramesh R Kozhaya L McKevitt K Djuretic IM Carlson TJ Quintero MA. Pro-inflammatory human Th17 cells selectively express P-glycoprotein and are refractory to glucocorticoids. J Exp Med. (2014) 211:89–104. 10.1084/jem.20130301 24395888 PMC3892977

[24] Wang C Yosef N Gaublomme J Wu C Lee Y Clish CB. CD5L/AIM Regulates Lipid Biosynthesis and Restrains Th17 *Cell* Pathogenicity. Cell. (2015) 163:1413–27. 10.1016/j.cell.2015.10.068 26607793 PMC4671820

[25] Uniken Venema WT Voskuil MD Vila AV van der Vries G Jansen BH Jabri B Single-Cell RNA sequencing of blood and ileal T cells from patients with crohn's disease reveals tissue-specific characteristics and drug targets. Gastroenterology. (2019) 156:812–5 e822. 10.1053/j.gastro.2018.10.046 30391472 PMC6759855

[26] Patil VS Madrigal A Schmiedel BJ Clarke J O'Rourke P de Silva AD. Precursors of human CD4^+^ cytotoxic T lymphocytes identified by single-cell transcriptome analysis. Sci Immunol. (2018) 3:eaan8664. 10.1126/sciimmunol.aan8664 29352091 PMC5931334

[27] Lee Y Awasthi A Yosef N Quintana FJ Xiao S Peters A. Induction and molecular signature of pathogenic TH17 cells. Nat Immunol. (2012) 13:991–9. 10.1038/ni.2416 22961052 PMC3459594

[28] Gerlach K Hwang Y Nikolaev A Atreya R Dornhoff H Steiner S. TH9 cells that express the transcription factor PU.1 drive T cell-mediated colitis via IL-9 receptor signaling in intestinal epithelial cells. Nat Immunol. (2014) 15:676–86. 10.1038/ni.2920 24908389

[29] Stanko K Iwert C Appelt C Vogt K Schumann J Strunk FJ. CD96 expression determines the inflammatory potential of IL-9-producing Th9 cells. Proc Natl Acad Sci USA. (2018) 115:E2940–9. 10.1073/pnas.1708329115 29531070 PMC5879650

[30] Nalleweg N Chiriac MT Podstawa E Lehmann C Rau TT Atreya R. IL-9 and its receptor are predominantly involved in the pathogenesis of UC. Gut. (2015) 64:743–55. 10.1136/gutjnl-2013-305947 24957265

[31] Kobayashi T Okamoto S Hisamatsu T Kamada N Chinen H Saito R. IL23 differentially regulates the Th1/Th17 balance in ulcerative colitis and Crohn's disease. Gut. (2008) 57:1682–9. 10.1136/gut.2007.135053 18653729

[32] Kleinschek MA Boniface K Sadekova S Grein J Murphy EE Turner SP. Circulating and gut-resident human Th17 cells express CD161 and promote intestinal inflammation. J Exp Med. (2009) 206:525–34. 10.1084/jem.20081712 19273624 PMC2699125

[33] Muranski P Borman ZA Kerkar SP Klebanoff CA Ji Y Sanchez-Perez L. Th17 cells are long lived and retain a stem cell-like molecular signature. Immunity. (2011) 35:972–85. 10.1016/j.immuni.2011.09.019 22177921 PMC3246082

[34] Oestreich KJ Huang AC Weinmann AS. The lineage-defining factors T-bet and Bcl-6 collaborate to regulate Th1 gene expression patterns. J Exp Med. (2011) 208:1001–13. 10.1084/jem.20102144 21518797 PMC3092354

[35] Zhang L Yu X Zheng L Zhang Y Li Y Fang Q. Lineage tracking reveals dynamic relationships of T cells in colorectal cancer. Nature. (2018) 564:268–72. 10.1038/s41586-018-0694-x 30479382

[36] Tang C Kakuta S Shimizu K Kadoki M Kamiya T Shimazu T Suppression of IL-17F, but not of IL-17A, provides protection against colitis by inducing Treg cells through modification of the intestinal microbiota. Nat Immunol. (2018) 19:755–65. 10.1038/s41590-018-0134-y 29915298

[37] Jaensson E Uronen-Hansson H Pabst O Eksteen B Tian J Coombes JL. Small intestinal CD103+ dendritic cells display unique functional properties that are conserved between mice and humans. J Exp Med. (2008) 205:2139–49. 10.1084/jem.20080414 18710932 PMC2526207

[38] Ahern PP Schiering C Buonocore S McGeachy MJ Cua DJ Maloy KJ. Interleukin-23 drives intestinal inflammation through direct activity on T cells. Immunity. (2010) 33:279–88. 10.1016/j.immuni.2010.08.010 20732640 PMC3078329

[39] Gaublomme JT Yosef N Lee Y Gertner RS Yang LV Wu C. Single-*cell* genomics unveils critical regulators of Th17 cell pathogenicity. Cell. (2015) 163:1400–12. 10.1016/j.cell.2015.11.009 26607794 PMC4671824

[40] Mazzoni A Maggi L Siracusa F Ramazzotti M Rossi MC Santarlasci V. Eomes controls the development of Th17-derived (non-classic) Th1 cells during chronic inflammation. Eur J Immunol. (2019) 49:79–95. 10.1002/eji.201847677 30144030

[41] Geginat J Paroni M Kastirr I Larghi P Pagani M Abrignani S. Reverse plasticity: TGF-beta and IL-6 induce Th1-to-Th17-cell transdifferentiation in the gut. Eur J Immunol. (2016) 46:2306–10. 10.1002/eji.201646618 27726139

[42] Maxwell JR Zhang Y Brown WA Smith CL Byrne FR Fiorino M. Differential roles for interleukin-23 and interleukin-17 in intestinal immunoregulation. Immunity. (2015) 43:739–50. 10.1016/j.immuni.2015.08.019 26431947

[43] Chapuy L Bsat M Sarkizova S Rubio M Therrien A Wassef E. Two distinct colonic CD14(+) subsets characterized by single-cell RNA profiling in Crohn's disease. Mucosal Immunol. (2019) 12:703–19. 10.1038/s41385-018-0126-0 30670762

[44] Sano T Huang W Hall JA Yang Y Chen A Gavzy SJ. An IL-23R/IL-22 circuit regulates epithelial serum amyloid A to promote local effector Th17 responses. Cell. (2015) 163:381–93. 10.1016/j.cell.2015.08.061 26411290 PMC4621768

[45] Chen L He Z Iuga AC Martins Filho SN Faith JJ Clemente JC. Diet modifies colonic microbiota and CD4(+) T-cell repertoire to induce flares of colitis in mice with myeloid-cell expression of interleukin 23. Gastroenterology. (2018) 155:1177–91.e1116. 10.1053/j.gastro.2018.06.034 29909020 PMC6174107

[46] Allocca M Furfaro F Fiorino G Gilardi D D'Alessio S Danese S Can IL-23 be a good target for ulcerative colitis? Best Pract Res Clin Gastroenterol. (2018) 32–33:95–102. 10.1016/j.bpg.2018.05.016 30060945

