# Supplementary material for: Differential Pathogenic Th17 Profile in Mesenteric Lymph Nodes of Crohn's Disease and Ulcerative Colitis Patients
Source: Front Immunol. 2019 May 28;10:1177. doi: 10.3389/fimmu.2019.01177 (PMC6547831; doi:10.3389/fimmu.2019.01177)
Supplement: Supplementary file 1 [file Data_Sheet_1.PDF]

Supplemental Table 1. Clinical information

	CD	UC
**N**	25	9
**Females, n (%)**	16 (64)	5 (55.5)
**Age, median (range)**	39 (24-67)	33 (18-80)
**Age at diagnosis**		
< 16	5	2
17-40	18	4
> 40	2	3
**Treatment**		
None	4	1
Thiopurine or methotrexate	15	3
TNFα inhibitor	11	2
Anti-IL-12p40	0	0
Anti-α4β7 integrin	1	2
5-ASA	2	
Corticosteroid	7	7
**Disease location - CD**		
Terminal ileum	1	
Colon	8	
Ileocolonic	16	
Perianal	7	
**Disease behavior - CD**		
Non-stricturing - Non-penetrating	0	
Stricturing	15	
Fistula	17	
Abscess	8	
**Disease location - UC**		
Proctitis		1
Left side colitis		1
Pancolitis		7
Proximal colitis		0

Supplemental Table 2. Anti-human antibodies.

Anti-human Antibody	Conjugate	Clone	Company
CCR6 (CD196)	PE	G034E3	Biolegend
CD25	APC	M-A251	BD Biosciences
CD25	BV605	BC96	Biolegend
CD3	BV510	UCHT1	Biolegend
CD3	BUV496	UCHT1	BD Biosciences
CD4	BV510	RPA-T4	Biolegend
CD4	BV785	OKT4	Biolegend
CD45RA	APC	HI100	Biolegend
CD45RA	APC FIRE	HI100	Biolegend
CD45RO	PerCP/Cy5.5	UCHL1	Biolegend
CD62L	PeCy7	DREG-56	Biolegend
CD62L	BV421	DREG-56	Biolegend
CD8	APC	RPA-T8	Biolegend
CD8	BUV737	SK1	BD Biosciences
CXCR3	Alexa Fluor 488	G025H7	Biolegend
CXCR3	BUV395	1C6	BD Biosciences
CXCR5	AF700	J252D4	Biolegend
FoxP3	APC	PCH101	eBioscience
ICOS (CD278)	BV421	C398.4A	Biolegend
IFN-gamma	PerCP/Cy5.5	4S.B3	Biolegend
IFN-gamma	BV711	4S.B3	Biolegend
IFN-gamma	BV421	4S.B3	Biolegend
IFN-gamma	AF700	4S.B3	Biolegend
IL17A	Alexa Fluor 647	BL168	Biolegend
IL17A	AF700	BL168	Biolegend
IL17A	BV421	BL168	Biolegend
Ki-67	BV711	Ki-67	Biolegend
TCR Vα4.24-Jα18	APC	6B11	Biolegend
TCR Vα7.2	PerCP/Cy5.5	3C10	Biolegend
TCR γ/δ	FITC	B1	Biolegend
ROR gamma (t)	APC	AFKJS-9	eBioscience
T-bet	FITC	4B10	Santa Cruz Biotechnology

Supplemental Table 3. Genes differentially expressed between activated Th17 cells from CD and UC (FDR value between 0.005 and 0.05)

Higher in CD	Higher in UC
APP	*AHR*
ARHGDIB	*BATF*
BCL2	*CTNNB1*
CD163	*EBI3*
CD3E	*ICOS*
CD40LG	*IFNGR1*
CD5	*IL1A*
CD53	*IL1R1*
CD9	*IL4R*
CSF1	*LIF*
CXCR6	*MX1*
DEFB4A	*PTK2*
ETS1	*SELL*
FCGR3A/B	*SLAMF1*
GFI1	*TIGIT*
HLA−DRB3	
ICOSLG	
IL16	
IL26	
IRAK2	
ITGA5	
ITGAX	
LCP2	
MBP	
MCL1	
PML	
PTPRC_all	
STAT5B	
TNFRSF4	

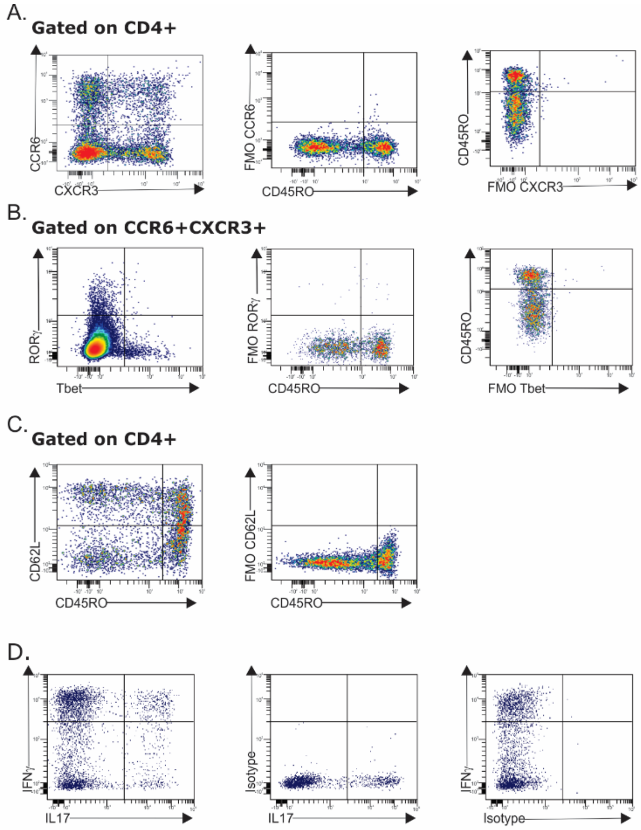

Supplementary Figure 1. Fluoresence minus one (FMO) and isotype-matched control antibodies .
(A and C) Surface staining on mLN from IBD patients.
(B) Intra-nuclear staining on mLN of IBD patients.
(D) $CCR6^{+}CXCR3^{+}$ TEM cells from CD donor were culture for 6 days with anti-CD3/CD28 beads. On the last day, PMA-ionomycin was added for 6 hours and Brefeldin A for the last 3 hours

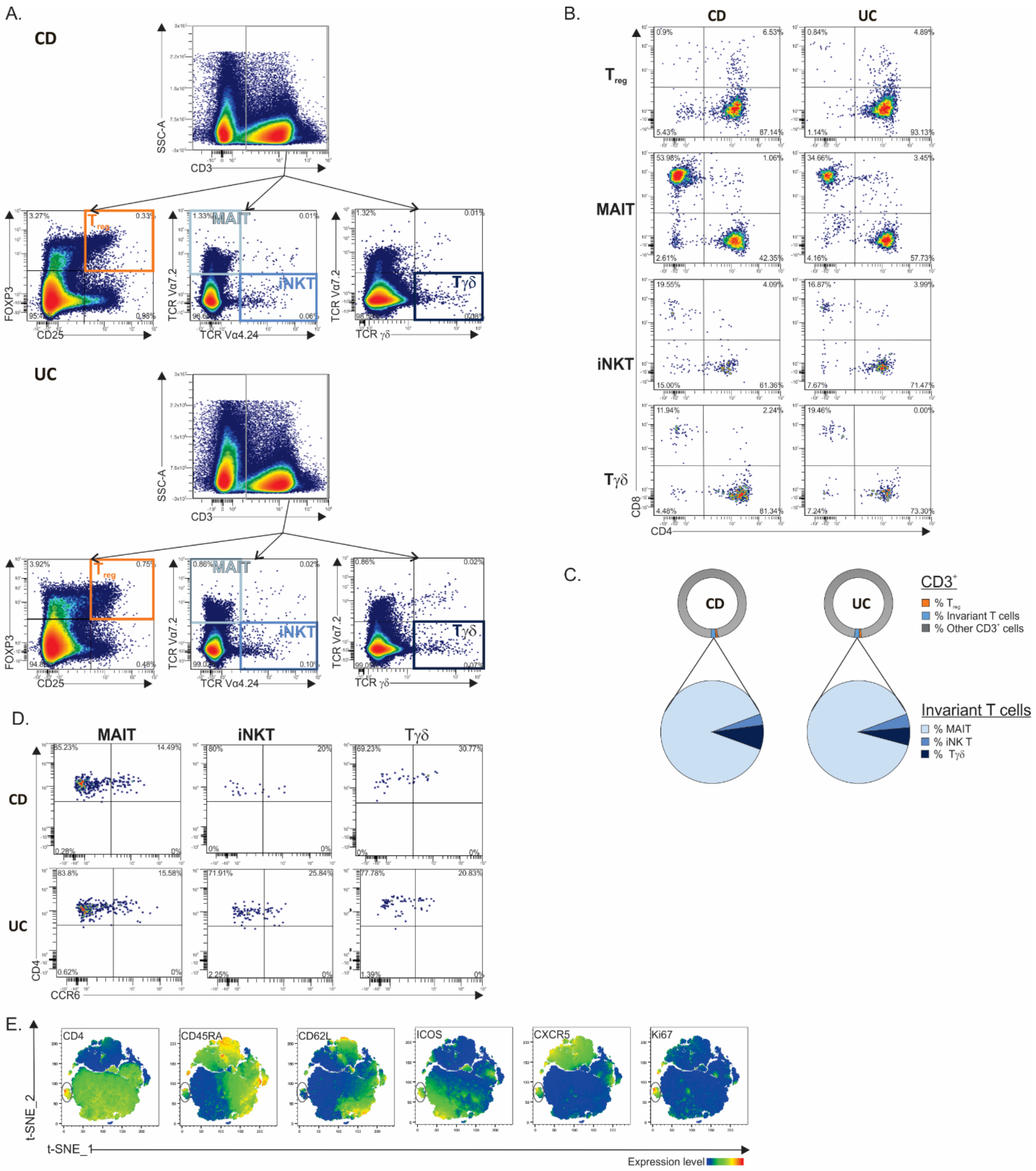

Supplementary Figure 2. Tfh, T_{reg} and invariant T cell populations in mLNs of IBD patients.

(A) Representative dot plots for identifying T_{reg} ($CD25^+Foxp3^+$), MAIT (TCR $V\alpha7.2^+$TCR $V\alpha4.24^-$), iNKT (TCR $V\alpha7.2^-$TCR $V\alpha4.24^+$) and γδ T ($TCR\gamma\delta^+$TCR $V\alpha7.2^-$) cells among $CD3^+$ T cells. (B) Representative dot plots showing CD4 and CD8 expression on various T cell subpopulations. (C) Pie chart representing the proportion of T cell subsets among $CD3^+$ T cells. (D) Representative dot plots showing CCR6 expression on invariant T cell populations. (E) $CD3^+$ T cells were concatenated for *t*-SNE analysis. Feature plots of the indicated antigens.

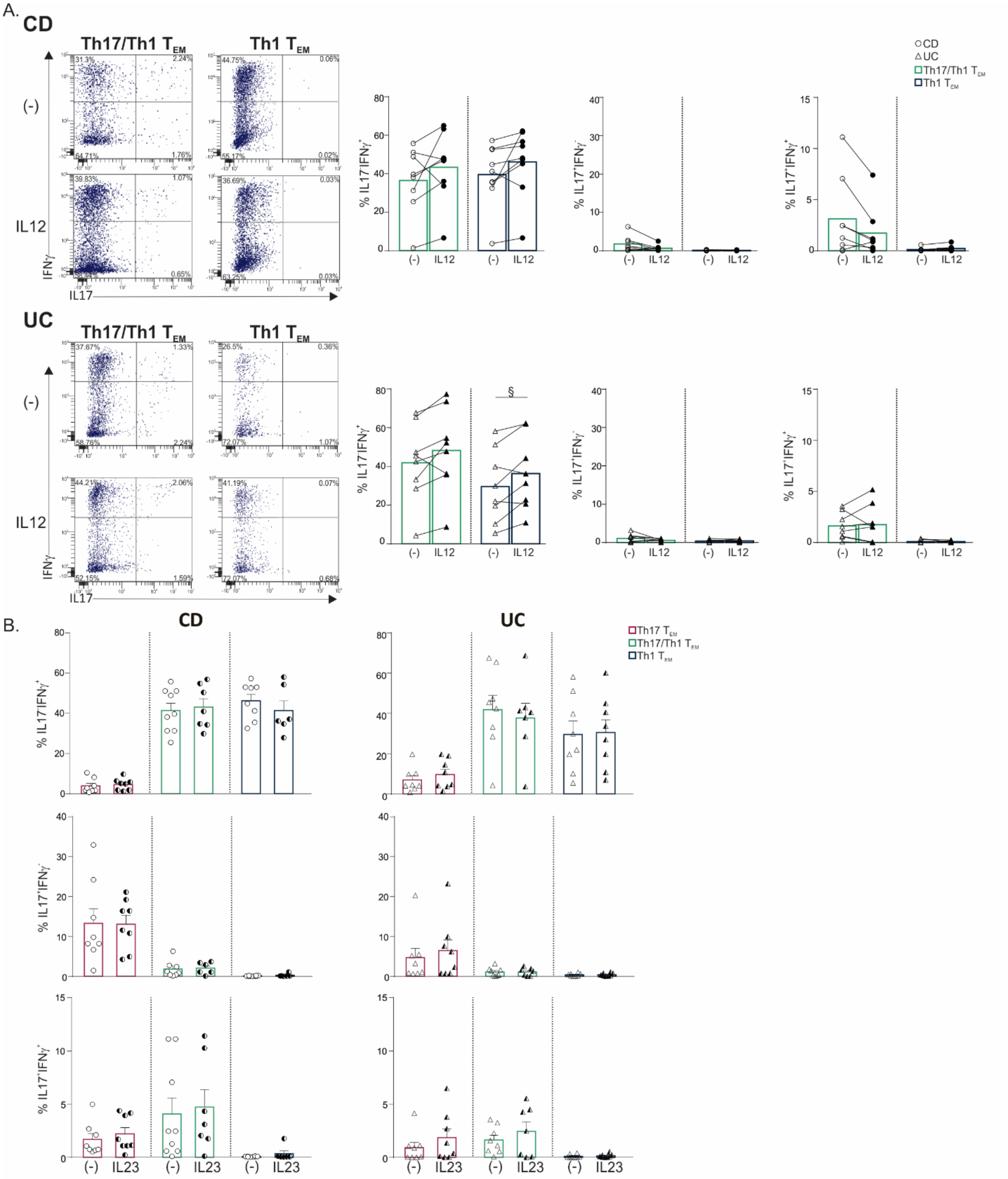

Supplementary Figure 3. Effect of IL12 and IL23 on Th17, Th17/Th1 and Th1 cells in mLNs of IBD patients.

Th T_{EM} cell subsets in mLNs of CD and UC were sorted according to the gating strategy shown in Figure 2A. Th T_{EM} cell subsets cultured with or without (A) IL12 or (B) IL23. Representative dot plots and frequencies of IL17 and IFNγ expression. Paired t-test (§).
